# 1041. *In vitro* activity of tebipenem against a recent collection of fastidious organisms recovered from respiratory tract infections

**DOI:** 10.1093/ofid/ofab466.1235

**Published:** 2021-12-04

**Authors:** S J Ryan Arends, Abby L Klauer, Nicole Cotroneo, Ian A Critchley, Rodrigo E Mendes

**Affiliations:** 1 JMI Laboratories, North Liberty, Iowa; 2 Spero Therapeutics

## Abstract

**Background:**

Tebipenem is under development as an oral treatment option for complicated urinary tract infections and acute pyelonephritis. This study further evaluated the *in vitro* activity of tebipenem against various fastidious organisms recovered from community-acquired respiratory tract infections (CARTIs).

**Methods:**

The study included a total of 2,476 fastidious organisms: *Haemophilus influenzae* (692 isolates, including fluoroquinolone-resistant, β-lactamase-positive, and β-lactamase-negative ampicillin-resistant [BLNAR]), *Haemophilus parainfluenzae* (30 isolates, including β-lactamase-positive and BLNAR), *Moraxella catarrhalis* (490 isolates), and *Streptococcus pneumoniae* (1,264 isolates, including penicillin-resistant). The isolates were collected primarily from CARTIs (90.8%) and pneumonia in hospitalized patients (PIHPs, 9.2%). Organisms were tested using reference broth microdilution methods in a central laboratory.

**Results:**

Tebipenem had MIC_90_ values of 0.5 mg/L against *H. influenzae* and 1 mg/L against *H. parainfluenzae* isolates. All 18 BLNAR isolates from these two species were inhibited at ≤1 mg/L of tebipenem. The MIC_90_ values observed for ertapenem and meropenem was 0.25 mg/L for these organisms. Tebipenem displayed good activity against *M. catarrhalis* (MIC_90_, 0.03 mg/L). Tebipenem inhibited 100% of *S. pneumoniae* isolates at ≤1 mg/L. Tebipenem activity (MIC_90_, 0.12 mg/L) was 8-fold greater than ertapenem (MIC_90_, 1 mg/L) against *S. pneumoniae* isolates.

**Conclusion:**

Tebipenem displayed potent activity against fastidious organisms causing respiratory tract infections. Greater than 99.7% of all *Haemophilus* isolates, including all BLNAR, were inhibited at ≤1 mg/L. All *M. catarrhalis* isolates were inhibited at ≤0.03 mg/L. Although tebipenem activity correlated with penicillin resistance, all *S. pneumoniae* isolates were inhibited at ≤1 mg/L. Tebipenem *in vitro* activity was greater than ertapenem when tested against *S. pneumoniae* isolates. This data supports the possible development of tebipenem as an oral option for combating CARTIs caused by these organisms.

Table

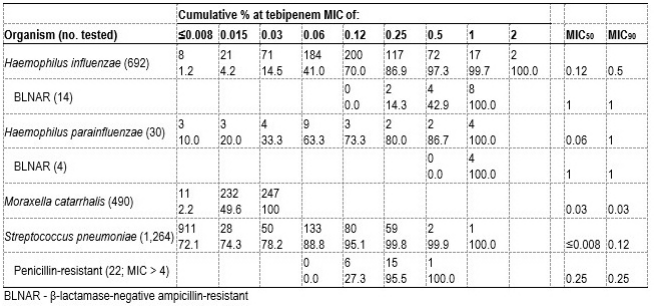

**Disclosures:**

**S J Ryan Arends, PhD**, **AbbVie (formerly Allergan**) (Research Grant or Support)**GlaxoSmithKline, LLC** (Research Grant or Support)**Melinta Therapeutics, LLC** (Research Grant or Support)**Nabriva Therapeutics** (Research Grant or Support)**Spero Therapeutics** (Research Grant or Support) **Abby L. Klauer, n/a**, **Cidara Therapeutics, Inc.** (Research Grant or Support)**Spero Therapeutics** (Research Grant or Support) **Nicole Cotroneo**, **Spero Therapeutics** (Employee, Shareholder) **Ian A. Critchley, Ph.D.**, **Spero Therapeutics** (Employee, Shareholder) **Rodrigo E. Mendes, PhD**, **AbbVie** (Research Grant or Support)**AbbVie (formerly Allergan**) (Research Grant or Support)**Cipla Therapeutics** (Research Grant or Support)**Cipla USA Inc.** (Research Grant or Support)**ContraFect Corporation** (Research Grant or Support)**GlaxoSmithKline, LLC** (Research Grant or Support)**Melinta Therapeutics, Inc.** (Research Grant or Support)**Melinta Therapeutics, LLC** (Research Grant or Support)**Nabriva Therapeutics** (Research Grant or Support)**Pfizer, Inc.** (Research Grant or Support)**Shionogi** (Research Grant or Support)**Spero Therapeutics** (Research Grant or Support)

